# Multimodal neuroimaging insights into the neurobiology of healthy aging across the lifespan

**DOI:** 10.1007/s00259-025-07100-w

**Published:** 2025-02-01

**Authors:** Laust Vind Knudsen, Tanja Maria Michel, Ziba Ahangarani Farahani, Manouchehr Seyedi Vafaee

**Affiliations:** 1 Department of Psychiatry, University of Southern Denmark, Odense University Hospital, Odense C, 5000 Denmark; 2 Department of Nuclear Medicine, Odense University Hospital, Odense C, 5000 Denmark

**Keywords:** PET, MRI, Glucose metabolism, Amyloid-beta, Functional connectivity, Aging

## Abstract

**Purpose:**

The purpose of this study was to advance our understanding of the neurobiology of healthy aging, which is crucial for improving quality of life and preventing age-related diseases. Despite its importance, a comprehensive investigation of this process has yet to be fully characterized.

**Methods:**

We used a hybrid PET/MRI scanner to assess neurophysiological parameters in 80 healthy individuals aged 20–78. Cerebral amyloid-beta (Aβ) deposition and glucose metabolism were assessed using PET scans, while participants underwent simultaneous MRI scans.

**Results:**

We found a positive correlation between Aβ-deposition and aging, and a negative correlation between glucose metabolism and aging. The insula showed the strongest negative correlation between glucose metabolism and age (Spearman’s *r* = -0.683, 95% CI [-0.79, -0.54], *p* < 0.0001), while the posterior cingulate cortex had the strongest positive correlation between Aβ-deposition and age (Spearman’s *r* = 0.479, 95% CI [0.28, 0.64], *p* < 0.0001). These results suggest a spatially dependent link between Aβ-deposition and metabolism in healthy older adults, indicating a compensatory mechanism in early Alzheimer’s. Additionally, Aβ-deposition was linked to changes in interregional neural communication.

**Conclusions:**

Our study confirms previous findings on aging and offers new insights, particularly on the role of Aβ-deposition in healthy aging. We observed a linear increase in Aβ-deposition, alongside decreases in white matter integrity, cerebral blood flow, and glucose metabolism. Additionally, we identified a complex regional relationship between Aβ-deposition, glucose metabolism, and neural communication, possibly reflecting compensatory mechanisms.

**Supplementary Information:**

The online version contains supplementary material available at 10.1007/s00259-025-07100-w.

## Introduction

Aging is a gradual process starting in adulthood, with bodily functions declining by middle age [1]. The brain, a key target, experiences reduced neurogenesis, mitochondrial issues, impaired metabolism, oxidative stress, and cognitive decline [2]. The reduction of neurotransmitters [3, 4] and increased amyloid-beta (Aß) plaques are key factors in aging and related neurological disorders [5]. Brain energy is mainly provided by glucose metabolism [6]. Molecular imaging studies show that while cerebral blood flow (CBF) may vary with age [7], cerebral metabolic rate of glucose (CMRglc) decline more significantly in disorders like Alzheimer’s disease (AD) than in healthy aging [8]. AD is a global healthcare issue, with cases expected to reach 153 million by 2050 due to aging populations [9]. The amyloid cascade hypothesis proposed by Hardy and Higgins [10], supported by genetic and imaging studies, suggests that AD results from abnormal Aβ-plaque buildup in the brain, leading to neurofibrillary tangles and cell death. Aβ-plaques may start accumulating 20 years before symptoms, followed by tau aggregates and neurodegeneration [11]. Significant levels of Aβ are found in healthy older individuals without evidence of clinically apparent cognitive decline [12], as a result imaging of Aβ-deposition could be a promising tool for early diagnosis and treatment assessment. The Pittsburgh Compound-B ([11 C]-PiB) is ideal for positron emission tomography (PET) to assess Aβ-deposition [13]. Imaging studies show early Aβ buildup in the cingulate, orbitofrontal, and precuneus regions of cognitively healthy older adults [14–16]. However, Aβ-deposition across the lifespan remains understudied, with only one study exploring it in healthy individuals of varying ages [5]. This study aimed to examine the relationship between age and Aβ-deposition, as well as its spatial distribution. Additionally, it investigated the effects of Aβ-deposition on CMRglc, CBF, functional connectivity (FC), and white matter integrity. The proposed studies aimed to test hypotheses linking mechanisms of brain aging, including: (1) Aβ-deposition increases with age while glucose metabolism declines, and these are inversely related; (2) Aβ affects both increased and decreased functional connectivity in the default mode network; (3) white matter integrity worsens with age and Aβ-deposition; (4) CBF declines with age and is negatively impacted by Aβ-deposition. To achieve this, PET, in conjunction with functional MRI (fMRI), diffusion MRI (dMRI), and arterial spin labeling (ASL), were used to measure and analyse these changes. This approach allowed us to measure and correlate changes in these variables in the aging brain, offering insights into their interactions during healthy aging and highlighting potential disruptions.

## Materials and methods

### Participants

Initially, 83 subjects were recruited through a company for research subject recruitment. The recruitment and scans were performed from January 2021 until September 2023. The scans were performed at the Nuclear Medicine department of Odense University Hospital in Denmark. Following an introductory telephone conversation outlining the scanning procedure, participants provided documentation from their personal physician confirming their neurological and psychiatric health status, otherwise they were excluded. Additionally, participants were excluded if they were using any prescription medication, except for over-the-counter remedies such as cold medicine or temporary pain relievers, if younger than 20 years, and if not fasted > 6 h before the scan. On the day of the scan, a physician conducted a brief health assessment for all participants to ensure their well-being. Two subjects were excluded from the study due to suspicious MRI scans. A total of 83 healthy participants underwent simultaneous PET and MRI scans. Among them, 81 PiB scans and 79 FDG ([2-[18 F]-fluoro-2-deoxy-D-glucose]) scans were without structural abnormalities and of adequate image quality. One subject (aged 70) showed unusually high Aβ deposition, approximately double that of age-matched individuals, and was excluded as an outlier using the Interquartile Range method. This exclusion was necessary because our study focuses on Aβ deposition in normal aging, and the elevated SUVR in this individual likely indicates early-stage AD. Consequently, the present study includes 80 participants (See Table in Online Resource 1).

### Data acquisition and image processing

**Fig. 1:**
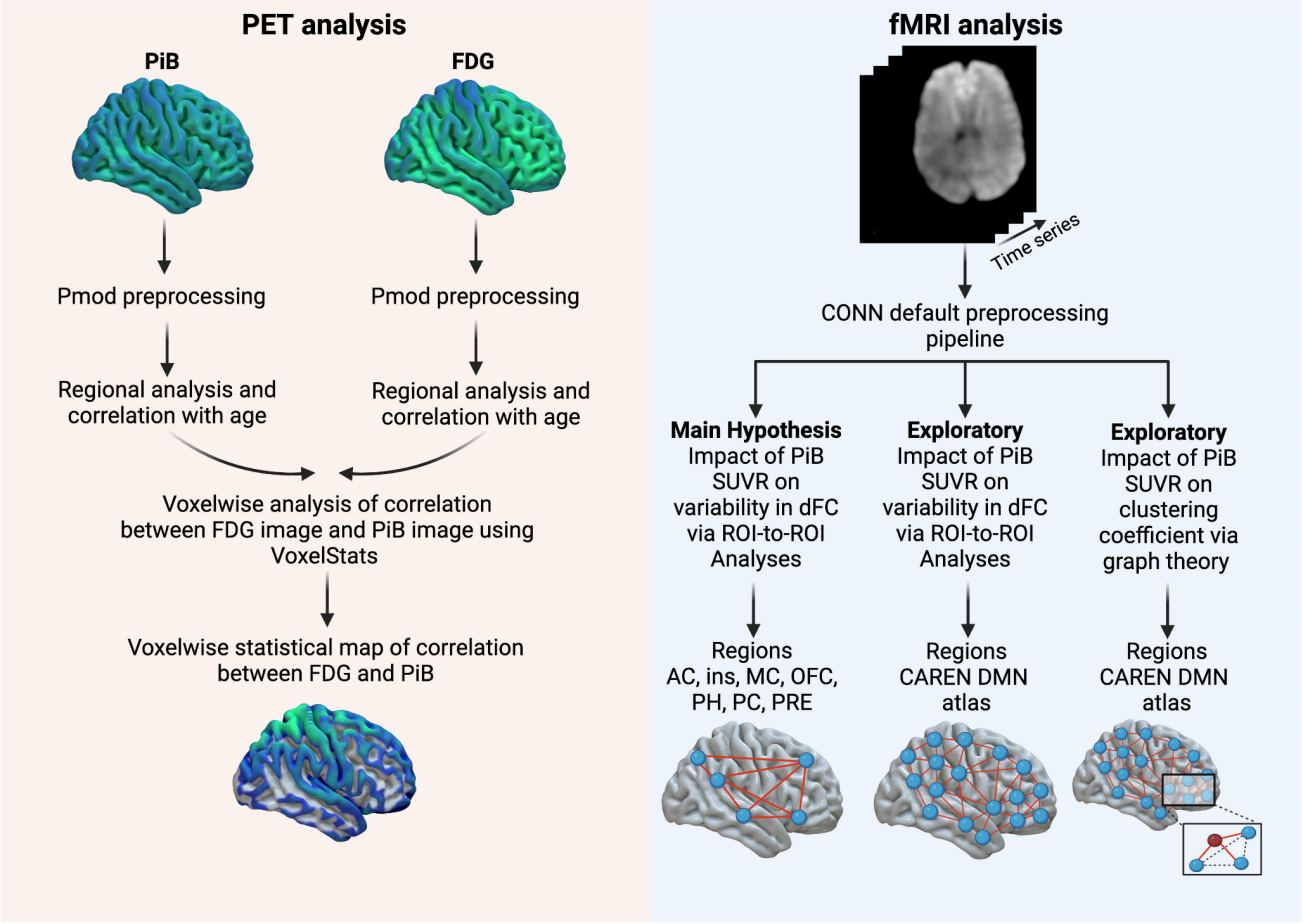
Schematic overview of the PET and fMRI analysis. Anterior Cingulate = AC. Insula = ins. Cingulate mid = MC. Precuneus = PRE. Posterior Cingulate = PC. Parahippocampus = PH. Orbito frontal cortex = OFC

Participants were scanned using a 3.0T GE Healthcare SIGNA™ PET/MR system (GE Healthcare, Chicago, IL, USA) equipped with a 19-channel head-and-neck unit. Attenuation-correction maps were generated using GE zero-echo-time based correction for PET/MRI brain imaging. Initially participants underwent a ^11^C-PiB PET scan. After a delay of five half-lives of this tracer, ^18^F-FDG was administered for the subsequent scan. Simultaneously structural T1-weighted MRI, fMRI, dMRI and ASL data were collected. Comprehensive details regarding the scanner acquisition parameters for PET and MRI are provided in Online Resource 2. Region-of-interest (ROI) analysis of the PET data was performed using PMOD software (PMOD Technologies Ltd., Version 4.4). Standardized uptake value ratio (SUVR) measures for both FDG and PiB were calculated by averaging unilateral time-activity curves (TACs) and normalizing them to the cerebellar grey matter TAC. For the PiB and FDG analysis, a composite mask was utilized, including the orbitofrontal cortex (OFC), insula, anterior cingulate cortex (AC), mid-cingulate cortex (MC), and posterior cingulate cortex (PC). A global mask was also applied, with details available in online resource 3. Additionally, voxel-wise analysis of the relationship between PiB- and FDG-SUVR, adjusted for age and gender, was conducted using VoxelStats [17]. For a detailed description of the PET analysis, refer to the Online Resource 4. The fMRI analysis was preprocessed and analyzed using the Functional Connectivity Toolbox (CONN version 22.a) in MATLAB R2021b [18], following the default CONN preprocessing pipeline. The ASL data were processed according to the method described by Zhao et al. [19]. and diffusion MRI data were analyzed using Tract-Based Spatial Statistics (TBSS) from the FSL software package [20]. Further details regarding the MRI analyses are provided in Online Resource 5, and a schematic overview is presented in Fig. 1.

### Statistical analysis

Regional FDG- and PiB-SUVR values were assessed for normality using the Kolmogorov-Smirnov test in GraphPad Prism 10.0.3 (GraphPad Inc.). Most regional PiB- and FDG-SUVR values were found to deviate from normal distribution (See table in Online Resource 6). Consequently, the Spearman correlation coefficient, as recommended by Kowalski et al. [21]. was employed to evaluate the correlation between age and FDG- and PiB-SUVR and subsequently the testing of statistical significance.

## Results

### Aβ-deposition

**Fig. 2:**
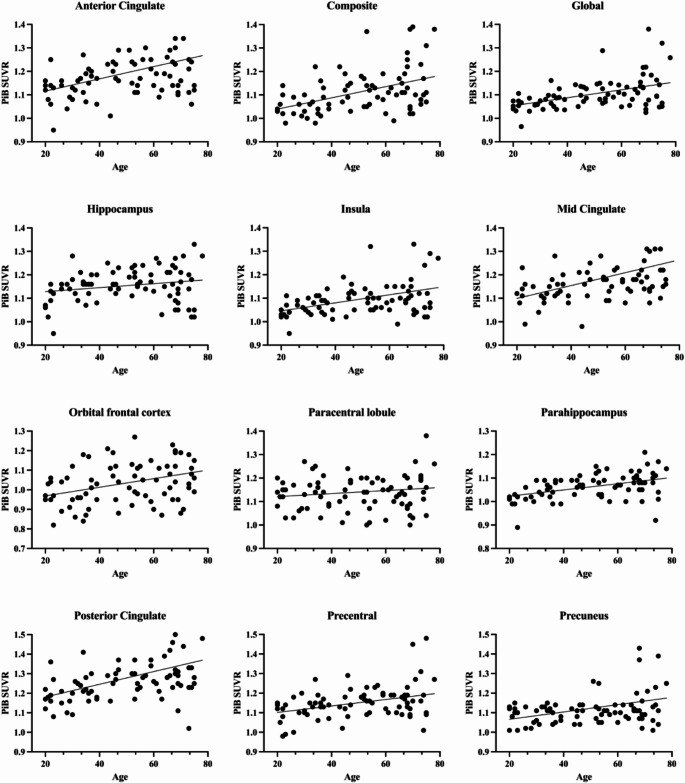
The correlation between Aβ-deposition and age

Initially, we explored the correlation between age and Aβ-deposition (Fig. 2). All included regions exhibited a positive correlation between Aβ-deposition and age, suggesting an accumulation of Aβ with increasing age (Table 1). The composite, global, MC, parahippocampus, and PC demonstrated the highest regional correlation coefficients, indicative of a moderate positive correlation strength (i.e. correlation coefficient > 0.4). In contrast, the remaining regions showed a statistically non-significant and weak positive correlation between Aβ-deposition and age, especially the hippocampus, paracentral lobule and precuneus.

### Glucose metabolism

**Fig. 3:**
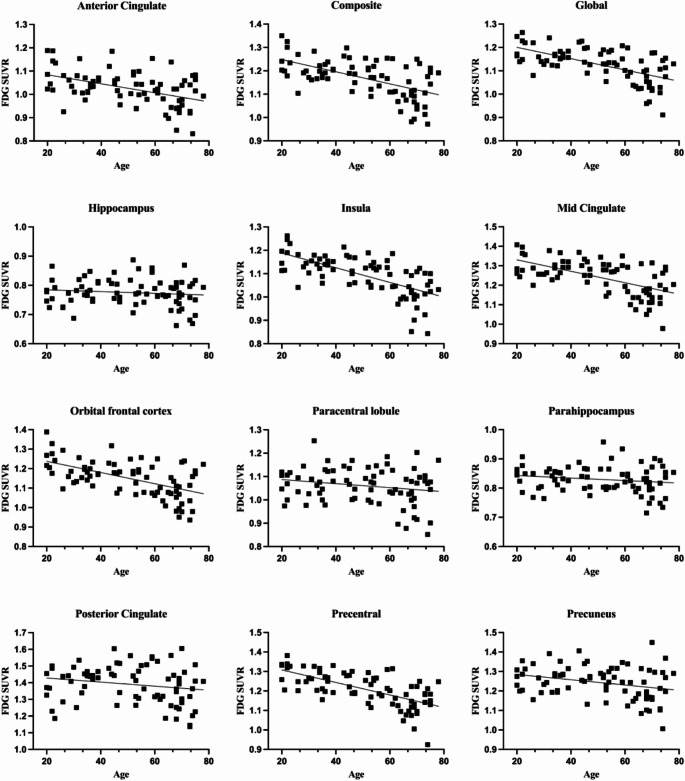
The correlation between glucose metabolism and age

Following this, we conducted an evaluation of the correlation between age and FDG-SUVR (Fig. 3). All regions exhibited a negative correlation between FDG-SUVR and age, implying a decline in glucose metabolism with increasing age (Table 1). Specifically, the AC, composite, global, insula, MC, OFC, and precentral gyrus demonstrated a moderate negative correlation with age. In contrast, the hippocampus, paracentral lobule, parahippocampus, PC, and precuneus showed a weak negative correlation strength.

**Table 1:** Median PiB-SUVR and FDG-SUVR, along with the corresponding Spearman correlation coefficient with age and p-value. Values are presented with 95% confidence interval in parentheses

Region	Median	Spearman *r*	*p*-value
**PiB**			
Anterior cingulate	1.170 (1.14 1.20)	0.375 (0.16 0.55)	0.0007
Composite	1.100 (1.07 1.12)	0.436 (0.23 0.60)	< 0.0001
Global	1.089 (1.08 1.11)	0.419 (0.21 0.59)	0.0001
Hippocampus	1.160 (1.14 1.17)	0.175 (-0.05 0.39)	0.1240
Insula	1.080 (1.06 1.10)	0.342 (0.12 0.53)	0.0020
Mid cingulate	1.160 (1.14 1.18)	0.466 (0.27 0.63)	< 0.0001
Orbitofrontal cortex	1.030 (0.98 1.06)	0.303 (0.08 0.49)	0.0066
Paracentral lobule	1.140 (1.12 1.16)	0.108 (-0.12 0.33)	0.3447
Parahippocampus	1.070 (1.05 1.08)	0.445 (0.24 0.61)	< 0.0001
Posterior cingulate	1.250 (1.23 1.29)	0.479 (0.28 0.64)	< 0.0001
Precentral gyrus	1.150 (1.13 1.16)	0.297 (0.07 0.49)	0.0080
Precuneus	1.110 (1.09 1.12)	0.225 (-0.01 0.43)	0.0462
**FDG**			
Anterior cingulate	1.023 (1.01 1.04)	-0.423 (-0.59 -0.22)	0.0001
Composite	1.179 (1.16 1.20)	-0.524 (-0.67 -0.33)	< 0.0001
Global	1.135 (1.12 1.15)	-0.580 (-0.71 -0.41)	< 0.0001
Hippocampus	0.773 (0.76 0.79)	-0.105 (-0.33 0.13)	0.3598
Insula	1.109 (1.06 1.13)	-0.683 (-0.79 -0.54)	< 0.0001
Mid cingulate	1.259 (1.23 1.28)	-0.533 (-0.68 -0.35)	< 0.0001
Orbitofrontal cortex	1.160 (1.13 1.18)	-0.510 (-0.66 -0.32)	< 0.0001
Paracentral lobule	1.071 (1.04 1.09)	-0.149 (-0.37 0.08)	0.1935
Parahippocampus	0.832 (0.81 0.85)	-0.166 (-0.38 0.07)	0.1470
Posterior cingulate	1.412 (1.36 1.44)	-0.225 (-0.43 0.04)	0.0479
Precentral gyrus	1.208 (1.186 1.25)	-0.664 (-0.77 -0.51)	< 0.0001
Precuneus	1.247 (1.21 1.28)	-0.260 (-0.46 0.03)	0.0215

### The influence of Aβ-deposition on glucose metabolism

**Fig. 4:**
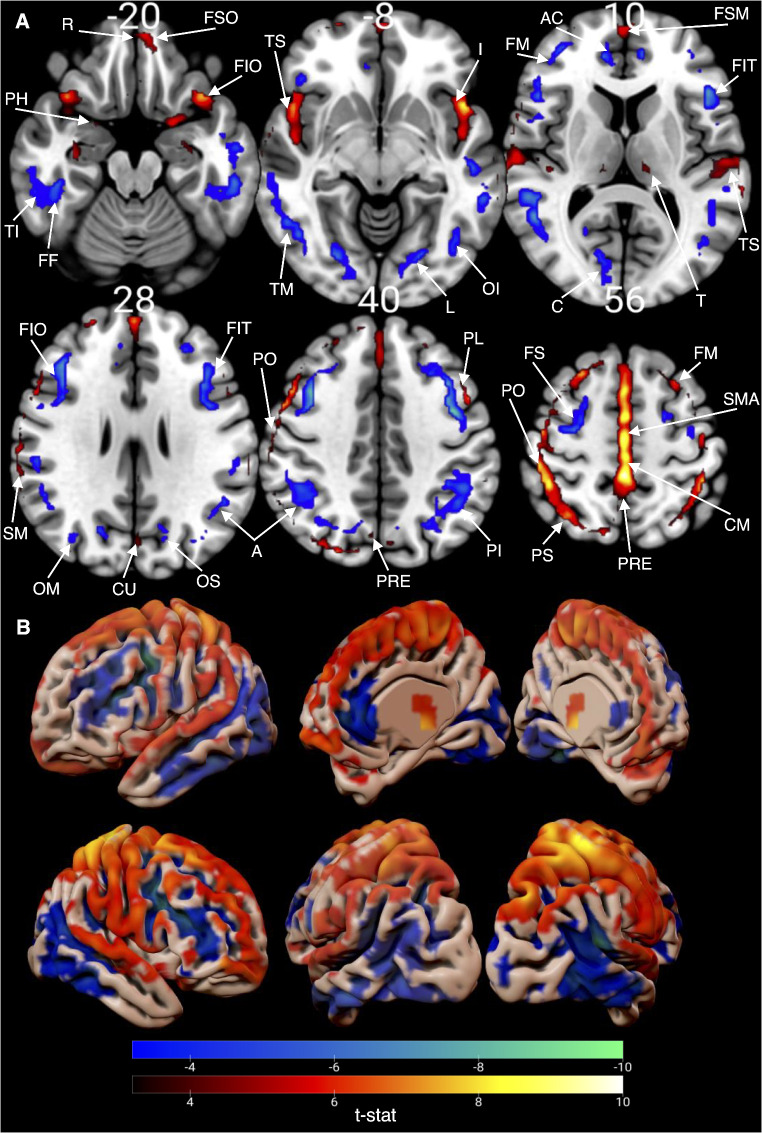
Correlation between glucose metabolism and Aβ-deposition. Significant multiple comparison corrected t-stat map depicting the voxel-wise correlation between PiB- and FDG-SUVR. The negative t-values displayed in blue-green demonstrate the statistically significant negative correlation between PiB- and FDG-SUVR. The positive t-values displayed in red-yellow demonstrate the positive correlation between PiB- and FDG-SUVR. **(A)** the statistical maps are presented in axial slices, and regions are marked based on the AAL atlas. **(B)** the statistical map is illustrated in surface-space for a clearer overview. Rectus = R. Frontal superior orbital = FSO. Parahippocampus = PH. Frontal inferior orbital = FSI. Temporal inferior = TI. Fusiform = FF. Temporal mid = TM. Frontal medial orbital = FMO. Anterior Cingulate = AC. Insula = I. Temporal superior = TS. Lingual = L. Occipital inferior = OI. Frontal superior medial = FSM. Frontal mid = FM. Frontal inferior tri = FIT. Calcarine Fissure = CF. Thalamus = T. Frontal inferior oper = FIO. Supramarginal = SM. Angular = A. Occipital Mid = OM. Cuneus = CU. Occipital superior = OS. Parietal inferior = PI. Parietal superior = PS. Precuneus = PRE. Frontal superior = FS. Cingulate mid = MC. Paracentral lobule = PL. Postcentral = PO. Supplementary motor area = SMA

After establishing the negative correlation of FDG-SUVR with age and the positive correlation of Aβ-deposition with age, both showing spatial dependence, we explored the correlation between PiB- and FDG-SUVR while correcting for age and gender. Voxel-wise analysis (Fig. 4) revealed regions with a negative relationship (Aβ increases as glucose metabolism decreases) and regions with a positive relationship (both Aβ and glucose metabolism increase). Regions showing significant correlations included the MC, OFC, insula, parahippocampus, and precuneus (positive correlation), and the AC and paracentral lobule (negative correlation) (Fig. 4). Generally, temporal regions showed mainly negative correlations, while occipital, frontal, and parietal regions exhibited positive correlations between Aβ and glucose metabolism.

### Influence of Aβ-deposition on brain connectivity

**Fig. 5:**
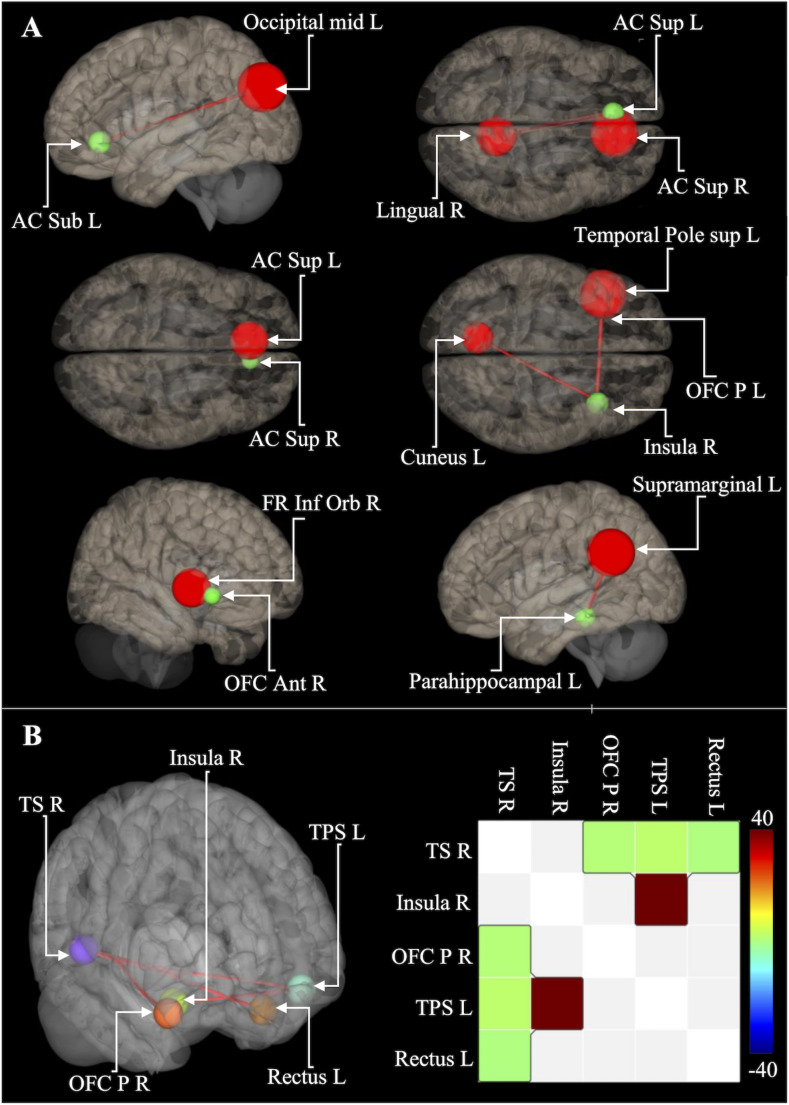
Results from the fMRI analysis. **(A)** Regions demonstrating a significant correlation between DMN Aβ-deposition and dFC variability. Green regions represent the seed-region, and the regions associated with changed dFC variability are marked in red. **(B)** Results from the analysis of all regions of the CAREN DMN atlas and related correlation matrix. The color scale represents the threshold-free-cluster-enhancement statistics. Anterior cingulate subgenual = AC sub. Anterior cingulate cortex Superior = AC sup. Orbito frontal cortex posterior = OFC post. Frontal inferior orbital = FR Inf Orb. Orbito frontal cortex anterior = OFC Ant. Temporal Superior R = TS R. Left = L. Right = R

We aimed to explore the links between Aβ-deposition and seed-based dFC within DMN regions to investigate Aβ-depositions impact on neural communication. We found a significant positive correlation between DMN Aβ-deposition and dFC variability from the AC, insula, OFC, and parahippocampus seed-regions (Fig. 5A), suggesting that increased Aβ-deposition in the DMN is linked to fluctuations in FC strength (Table in Online Resource 7). An exploratory analysis of all DMN regions, according to the CAREN DMN atlas [22], reaffirmed these findings, identifying a cluster comprising the insula, OFC, rectus, superior temporal, and temporal pole (cluster p-FDR = 0.02477) (Fig. 5B and Online Resource 8). Further analysis showed a significant positive correlation between the clustering coefficient and DMN Aβ-deposition in the left insula (p-FDR = 0.00076), indicating that higher Aβ-deposition increases the insula’s interconnectivity with adjacent structures (Online Resource 9).

### Age and Aβ-depositions influence on FA

We initially explored whether FA decreases with age. As anticipated, the results revealed widespread decreases in FA with advancing age (Online Resource 10), indicating a negative correlation between FA and age. Subsequently, we examined whether Aβ was associated with decreases in FA while adjusting for gender and age. However, this analysis yielded no significant results (nearest *p* = 0.274).

### Age and Aβ-depositions influence on CBF

We also investigated the relationship between age and CBF using ASL MRI. As expected, the findings revealed brain-wide decreases in CBF with advancing age (Online Resource 11), suggesting a negative correlation between CBF and age. Subsequently, we assessed the influence of Aβ on CBF, while controlling for gender and age. Nevertheless, the analysis produced non-significant findings, with the closest observed p-value being 0.260 located in the right precuneus.

## Discussion

The present study provides both confirmatory evidence and novel insights, enriching the current understanding in the field. First, we expand on the rare scientific documentation concerning Aβ-deposition in healthy aging. We demonstrate that global Aβ-deposition increases linearly with age and prove that this accumulation is spatially dependent. Moreover, we also confirm that global glucose metabolism decreases with age and that is also spatially dependent. Additionally, we illustrate an interesting and complex relationship between cerebral Aβ-deposition and glucose metabolism, where some brain regions exhibit positive while other regions demonstrate negative correlations. Furthermore, we demonstrate that Aβ-deposition is not only related to glucose metabolism but also neural communication between brain structures. Lastly, we shed light on the state of cerebral blood flow during the process of healthy aging.

### Correlation between Aβ-deposition and age

Our findings align with existing literature on Aβ-deposition across a broad age range of healthy controls. Rodrigue et al. [5] reported a significant positive correlation between age and Aβ-deposition, especially in the precuneus, PC, temporal cortex, and AC. Our study shows similar trends in the AC, PC, and to a lesser extent, the precuneus. Additional analysis revealed that Aβ-depositions in the PC, MC, parahippocampus, and global/composite masks positively correlate with age. Our correlation coefficients are close to Rodrigue et al.‘s, except for the precuneus (0.225 vs. 0.41). We believe this discrepancy is due to Rodrigue et al.‘s inclusion of Aβ-positive subjects, all of whom are over 70 years old, which likely skews the correlation upward. Oppositely, we excluded one subject with abnormally high SUVRs using the Interquartile Range method for outlier detection, which may account for the slight disparity.

### Correlation between glucose metabolism and age

The FDG-SUVR results demonstrated a negative correlation between cerebral glucose metabolism and age, consistent with existing cross-sectional [23]and longitudinal [24] studies linking decreased glucose metabolism to aging. Our findings mirror this, with the AC, OFC, and precentral gyrus showing strong negative correlations, while posterior regions like the precuneus and PC demonstrate weaker correlations. We also found a strong negative correlation in the insula, which has not been examined in prior studies. However, recent research suggests the insula is an early site of Aβ-deposition [14, 16], indicating it may be sensitive to age-related metabolic changes.

### Correlation between glucose metabolism and Aβ-deposition

We documented a negative correlation between PiB- and FDG-SUVR during healthy aging. Since aging is a risk factor for mild cognitive impairment (MCI) and AD, these findings may help understand AD progression. Both decreases and increases in brain glucose metabolism have been observed in early AD stages. For example, a study on subjective cognitive decline (SCD) patients, the earliest part of the AD continuum, showed hypometabolism in the right middle temporal gyrus (RMTG) and hypermetabolism in occipital regions compared to healthy controls [25]. The voxel-wise analysis of the correlation between PiB-SUVR and FDG-SUVR revealed similar spatial patterns. Specifically, the temporal regions showed predominantly negative correlations, indicating that higher PiB-SUVR levels were associated with lower FDG-SUVR levels. In contrast, the occipital and parietal regions primarily exhibited positive correlations (Fig. 4). Given the correlation between SCD and elevated Aβ-deposition [26], it is plausible that Aβ-deposition causes hypermetabolism in early AD stages or aging. Similarly, individuals in advanced AD stages also demonstrate hypermetabolism [27–29]. In a study by Ashraf et al. [28] four Aβ-negative and one Aβ-positive individual with MCI showed hypermetabolism in occipital, parietal areas, insula, and postcentral regions. Unlike other MCI individuals who exhibited hypometabolism and progressed to AD, these hypermetabolic subjects did not convert to AD within 18 months and had Aβ-deposition resembling elderly controls. This suggests that hypermetabolism may act as a compensatory mechanism against Aβ-deposition. The brain might increase glucose metabolism to counteract Aβ neurotoxicity, delaying cognitive decline. This hypothesis is supported by another study where healthy elderly subjects with increased Aβ-deposition in the precuneus showed hypermetabolism and improved verbal episodic memory, indicating that hypermetabolism helps preserve cognitive function temporarily [30].

### Correlation between Aβ-deposition and brain connectivity

The correlation between increased metabolic activity and excessive Aβ-deposition aligns with fMRI studies showing a positive relationship between Aβ-deposition and neural activation during episodic memory tasks [31]. Studies have found that Tau protein pathology is linked to hypoconnectivity, while Aβ-deposition is associated with increased FC [32–35]. Foster et al. [34] demonstrated that Aβ-deposition levels directly influenced whether hyperconnectivity or hypoconnectivity was observed in the DMN, with healthy controls showing hyperactivation at slightly elevated Aβ-deposition and hypoactivation at higher levels. Our results demonstrated a positive correlation between Aβ-deposition in the DMN and dFC variability in multiple regions. Enhanced dFC variability, defined as fluctuations in FC between regions, may indicate connectivity reorganization as a compensatory mechanism in defense against Aβ [35]. We also found an increased clustering coefficient in the insula, suggesting heightened local connectivity strength in response to Aβ-deposition. This local hyperconnectivity, interpreted as brain plasticity after neural network damage [36], may serve as a compensatory mechanism to uphold cognitive function. Overall, our results demonstrate a link between Aβ-deposition and alterations in neural communication within the DMN, affecting both FC variability and local neural communication.

### Correlation between FA, age and Aβ-deposition

We observed a pronounced and widespread correlation, between decreasing FA and advancing age, consistent with existing literature [37]. In contrast, no significant correlation was found between FA and Aβ-deposition in our study. While previous research [38] identified such a correlation in cognitively normal elderly individuals with varying Aβ-deposition, the relatively low Aβ-deposition and limited number of elderly subjects in our cohort may explain the absence of a significant correlation.

### Correlation between CBF, age and Aβ-deposition

While our study confirmed a significant and extensive correlation between declining CBF and increasing age, consistent with prior research [39], we did not observe a significant relationship between CBF and Aβ-deposition. Previous studies have documented that Aβ-deposition correlates with increased CBF in various brain regions, such as the insula, caudate, hippocampus, amygdala, frontal, and temporal regions, particularly among elderly Aβ-positive healthy controls [40, 41] (while AD is associated with lower CBF). It is plausible that these findings could have been replicated in our study with a larger cohort, allowing for more comprehensive acquisition of ASL data.

### Limitations

The current study has certain limitations. Firstly, PiB exhibits non-specific binding, including to cerebrovascular amyloid and white matter [42, 43], which complicates PET imaging. Despite this, PiB remains the gold standard for amyloid imaging due to its relatively low non-specific binding [44–47]. Secondly, at the start of our study, 17 subjects underwent a battery of neuropsychological and cognitive tests, including the Mini-Mental State Examination (MMSE), Clinical Dementia Rating (CDR), Alzheimer’s Disease Assessment Scale–Cognitive Subscale (ADAS-cog), and The Montreal Cognitive Assessment (MoCA). However, these tests were discontinued due to their length, which caused fatigue and discomfort among participants during the approximately three-hour scanning procedure. This led to movement-related artifacts and, in some cases, early withdrawal. Logistical constraints also prevented participants from returning for follow-up cognitive assessments. Importantly, none of the 17 subjects showed abnormal cognitive scores, as all were referred to us as healthy by their physicians. We believe these limitations do not significantly bias our results for several reasons. First, all participants were assessed as neurologically and psychiatrically healthy, based on information from their personal physicians and brief evaluations by our departmental physician, which included a conversation about the participants’ medical history and medication use, as well as an inspection of scans to confirm the absence of irregularities. Additionally, cognitive impairments are more commonly associated with tau pathology in the middle and later stages of the AD continuum [11, 48–50], making it unlikely that early-stage cognitive issues would be present in this cohort.

## Conclusions

Our study reveals a novel correlation between Aβ-deposition and hypermetabolism in healthy elderly controls, a phenomenon linked to early AD stages. This Aβ-deposition is correlated with alterations in neuronal activity (metabolism) and neural communication, possibly serving as a compensatory mechanism to maintain cognitive function. The insula showed the strongest negative correlation between glucose metabolism and age, indicating its vulnerability to aging. We also found a positive correlation between glucose metabolism and Aβ-deposition in the insula, supported by fMRI results showing altered dFC variability. The insula was the only region with a notable correlation between Aβ-deposition and clustering coefficient, suggesting a compensatory mechanism to maintain its sensory, emotional, motivational, and cognitive functions. Collectively, our results suggest the insula is particularly susceptible to aging and Aβ-deposition in the early phase of the AD continuum, playing a more significant role in aging and AD development than previously recognized.

## Electronic supplementary material

Below is the link to the electronic supplementary material.

Supplementary Material 1

Supplementary Material 2

Supplementary Material 3

Supplementary Material 4

Supplementary Material 5

Supplementary Material 6

Supplementary Material 7

Supplementary Material 8

Supplementary Material 9

Supplementary Material 10

Supplementary Material 11

## Data Availability

The data from this study are currently stored in a secure database owned by the University of Southern Denmark. This data may be shared with qualified investigators for non-commercial purposes, subject to restrictions regarding participant consent and data protection legislation.
