# Supplementary material for: Multimodal neuroimaging insights into the neurobiology of healthy aging across the lifespan
Source: Eur J Nucl Med Mol Imaging. 2025 Feb 1;52(7):2267–78. doi: 10.1007/s00259-025-07100-w (PMC12119650; doi:10.1007/s00259-025-07100-w)
Supplement: Supplementary file 1 — Supplementary Material 1 [file 259_2025_7100_MOESM1_ESM.docx]

**Multimodal Neuroimaging Insights into the Neurobiology of Healthy Aging Across the Lifespan**

European Journal of Nuclear Medicine and Molecular Imaging

Laust Vind Knudsen^1^, Tanja Maria Michel^1^**^†^**, Ziba Ahangarani Farahani^2^, Manouchehr Seyedi Vafaee^1,2^

**^†^**Shared first author

**Author affiliations:**

^1^ Department of Psychiatry, University of Southern Denmark, 5000 Odense C, Denmark

^2^ Department of Nuclear Medicine, Odense University Hospital, 5000 Odense C, Denmark

**Correspondence to:**
Manouchehr Seyedi Vafaee

University of Southern Denmark, J.B. Winsløws vej 18, 5000 Odense C, Denmark

E-mail: [mvafaee@health.sdu.dk](mailto:mvafaee@health.sdu.dk)

**Online Resource 1.** Participant and tracer characteristics for the study participants.

|  | **Mean** | **Min** | **Max** | **Range** | **SD** |  |
| --- | --- | --- | --- | --- | --- | --- |
| Age (years) | 50.52 | 20 | 78 | 58 | 17.88 |  |
| PiB Dose (MBq) | 531.7 | 269.6 | 702.2 | 432.5 | 90.96 |  |
| FDG Dose (MBq) | 305.4 | 197.0 | 408.0 | 211.0 | 54.78 |  |
| BMI (kg/m^2^) | 25.56 | 17.86 | 35.58 | 17.72 | 3.78 |  |

BMI = body mass index. SD = Standard deviation. PiB = Pittsburgh Compound-B. FDG = fluoro-2-deoxy-D-glucose. MBq = megaBecquerel.
