# Supplementary material for: Multimodal neuroimaging insights into the neurobiology of healthy aging across the lifespan
Source: Eur J Nucl Med Mol Imaging. 2025 Feb 1;52(7):2267–78. doi: 10.1007/s00259-025-07100-w (PMC12119650; doi:10.1007/s00259-025-07100-w)
Supplement: Supplementary file 2 — Supplementary Material 2 [file 259_2025_7100_MOESM2_ESM.docx]

**Multimodal Neuroimaging Insights into the Neurobiology of Healthy Aging Across the Lifespan**

European Journal of Nuclear Medicine and Molecular Imaging

Laust Vind Knudsen^1^, Tanja Maria Michel^1^**^†^**, Ziba Ahangarani Farahani^2^, Manouchehr Seyedi Vafaee^1,2^

**^†^**Shared first author

**Author affiliations:**

^1^ Department of Psychiatry, University of Southern Denmark, 5000 Odense C, Denmark

^2^ Department of Nuclear Medicine, Odense University Hospital, 5000 Odense C, Denmark

**Correspondence to:**
Manouchehr Seyedi Vafaee

University of Southern Denmark, J.B. Winsløws vej 18, 5000 Odense C, Denmark

E-mail: [mvafaee@health.sdu.dk](mailto:mvafaee@health.sdu.dk)

**Online resource 2.** Details concerning the PET and MRI acquisition.

All participants were scanned on a 3.0T GE Healthcare SIGNA^TM^ PET/MR (GE Healthcare, Chicago, IL, USA) system equipped with a 19-channel head-and-neck unit. PET images were reconstructed utilizing the Bayesian penalized-likelihood iterative image reconstruction algorithm (Q-clear) with a beta-value of 400. As proposed by Teoh *et al*.(1) this approach enhances image quality compared to the ordered-subset expectation maximization technique. Attenuation-correction maps were obtained through GE zero-echo-time (ZTE)-based attenuation correction, specifically designed for integrated PET/MRI brain imaging. We performed ^11^C-PiB PET scans for measurement of Aβ-plaque deposition. Intravenous injection of a mean dose of 531.7 (± 90.96) megabecquerel (MBq) was administered, followed by an acquisition in list-mode lasting 70 minutes from the initiation of the injection. Images were reconstructed in 38 frames: 12×5s, 6×10s, 3×20s, 4×30s, 5×60s, 4×5m, and 4×10m. FDG-SUVR was measured by the intravenous injection of a mean dose of 305.4 MBq (± 54.78) ^18^F-FDG. Subjects were fasted overnight and demonstrated a mean blood glucose of 5.43 ± 0.53 mmol/L. FDG PET list-mode acquisition continued for 60 minutes from the initiation of the injection and was reconstructed in 37 frames: 12×5s, 6×10s, 3×20s, 4×30s, 5×60s, 4×5m, and 3×10m. PiB and FDG images were reconstructed with a voxel size of 2.34×2.34×2.78 mm^3^ using a 128×128 matrix and 89 volume slices. T1-weighted MRI scans (3D FSPGR BRAVO) were conducted with an echo time (TE) of 2.3 ms and a repetition time (TR) of 6.5 ms. These scans utilized a 256×256 matrix and were reconstructed in a 512×512 matrix, producing a voxel size of 0.5×0.5×0.5 mm^3^. Functional MRI (fMRI) data (greEPI fMRI) with a TE of 30 ms and TR of 2000 ms were acquired and reconstructed in a 64×64 matrix with 40 volume slices, producing a voxel size of 3.75×3.75×4.5 mm^3^. fMRI data were collected over a 270-second period. Pseudo-continuous ASL data were obtained using a post-labeling delay of 2500 ms, TE of 9.312 ms, TR of 5386 ms, labeling duration of 1450 ms, and 4 number of excitations. The acquisition utilized a 3D readout with background suppression. The ASL images were captured in a 64x64 matrix with a FOV of 220 mm and a slice thickness of 3.5 mm. Subsequently, the images were reconstructed in a 128x128 matrix. The dMRI scan was performed in a 120x120 matrix with a 256 mm FOV and a slice thickness of 2 mm. The TE was 102 ms, and the TR was 1700 ms. The dMRI acquisition comprised 16 diffusion directions.

1. Teoh EJ, McGowan DR, Macpherson RE, Bradley KM, Gleeson F V. Phantom and clinical evaluation of the Bayesian penalized likelihood reconstruction algorithm Q.Clear on an LYSO PET/CT system. Journal of Nuclear Medicine. 2015;56(9):1447–52.
