# Supplementary material for: Multimodal neuroimaging insights into the neurobiology of healthy aging across the lifespan
Source: Eur J Nucl Med Mol Imaging. 2025 Feb 1;52(7):2267–78. doi: 10.1007/s00259-025-07100-w (PMC12119650; doi:10.1007/s00259-025-07100-w)
Supplement: Supplementary file 3 — Supplementary Material 3 [file 259_2025_7100_MOESM3_ESM.docx]

**Multimodal Neuroimaging Insights into the Neurobiology of Healthy Aging Across the Lifespan**

European Journal of Nuclear Medicine and Molecular Imaging

Laust Vind Knudsen^1^, Tanja Maria Michel^1^**^†^**, Ziba Ahangarani Farahani^2^, Manouchehr Seyedi Vafaee^1,2^

**^†^**Shared first author

**Author affiliations:**

^1^ Department of Psychiatry, University of Southern Denmark, 5000 Odense C, Denmark

^2^ Department of Nuclear Medicine, Odense University Hospital, 5000 Odense C, Denmark

**Correspondence to:**
Manouchehr Seyedi Vafaee

University of Southern Denmark, J.B. Winsløws vej 18, 5000 Odense C, Denmark

E-mail: [mvafaee@health.sdu.dk](mailto:mvafaee@health.sdu.dk)

**Online Resource 3.** Regions included in the global and composite mask. All regions are from the AAL3 atlas.

| Brain regions included in the global mask | | |
| --- | --- | --- |
| Precentral_L | Insula_L | SupraMarginal_L |
| Precentral_R | Insula_R | SupraMarginal_R |
| Frontal_sup_L | Cingulate_mid_L | Angular_L |
| Frontal_sup_R | Cingulate_mid_R | Anguar_R |
| Frontal_mid_L | Cingulate_post_L_ | Precuneus_L |
| Frontal_mid_R | Cingulate_post_R | Precuneus_R |
| Frontal_Inf_Oper_L | Hippocampus_L | Paracentral_Lobule_L |
| Frontal_Inf_Oper_R | Hippocampus_R | Paracentral_Lobule_R |
| Frontal_Inf_Tri_L | Parahippocampus_L | Caudate_L |
| Frontal_Inf_Tri_R | Parahippocampus_R | Caudate_R |
| Frontal_Inf_Orb_L_ | Amygdala_L | Putamen_L |
| Frontal_Inf_Orb_R | Amygdala_R | Putamen_R |
| Rolandic_Oper_L | Calcarine_L | Pallidum_L |
| Rolandic_Oper_R | Calcarine_R | Pallidum_R |
| Supp_Motor_area_L | Cuneus_L | Heschl_L |
| Supp_Motor_area_R | Cuneus_R | Heschl_R |
| Olfactory_L | Lingual_L | Temporal_Sup_L |
| Olfactory_R | Lingual_R | Temporal_Sup_R |
| Frontal_Sup_Medial_L | Occipital_Sup_L | Temporal_Pole_Sup_L |
| Frontal_Sup_Medial_R | Occipital_Sup_R | Temporal_Pole_Sup_R |
| Frontal_Med_Orb_L | Occipital_mid_L | Temporal_Mid_L |
| Frontal_Med_Orb_R | Occipital_mid_R | Temporal_Mid_R |
| Rectus_L | Occipital_Inf_L | Temporal_Pole_Mid_L |
| Rectus_R | Occipital_Ind_R | Temporal_Pole_Mid_R |
| OFC_Med_L | Fusiform_L | Temporal_Inf_L |
| OFC_med_R | Fusiform_R | Temporal_Inf_R |
| OFC_ant_L | Postcentral_L | ACC_Sub_L |
| OFC_ant_R | Postcentral_R | ACC_Sub_R |
| OFC_post_L | Parietal_Sup_L | ACC_Pre_L |
| OFC_post_R | Parietal_Sup_R | ACC_Pre_R |
| OFC_lat_L | Parietal_Inf_L | ACC_Sup_L |
| OFC_lat_R | Parietal_Inf_R | ACC_Sup_R |
|  |  |  |
| Brain regions included in the composite mask | | |
| OFC_med_L | OFC_lat_L | Cingulate_mid_L |
| OFC_med_R | OFC_lat_R | Cingulate_mid_R |
| OFC_ant_L | Insula_L | Cingulate_post_L_ |
| OFC_ant_R | Insula_R | Cingulate_post_R |
| OFC_post_L | Cingulate_ant_L |  |
| OFC_post_R | Cingulate_ant_R |  |
