# Supplementary material for: Multimodal neuroimaging insights into the neurobiology of healthy aging across the lifespan
Source: Eur J Nucl Med Mol Imaging. 2025 Feb 1;52(7):2267–78. doi: 10.1007/s00259-025-07100-w (PMC12119650; doi:10.1007/s00259-025-07100-w)
Supplement: Supplementary file 4 — Supplementary Material 4 [file 259_2025_7100_MOESM4_ESM.docx]

**Multimodal Neuroimaging Insights into the Neurobiology of Healthy Aging Across the Lifespan**

European Journal of Nuclear Medicine and Molecular Imaging

Laust Vind Knudsen^1^, Tanja Maria Michel^1^**^†^**, Ziba Ahangarani Farahani^2^, Manouchehr Seyedi Vafaee^1,2^

**^†^**Shared first author

**Author affiliations:**

^1^ Department of Psychiatry, University of Southern Denmark, 5000 Odense C, Denmark

^2^ Department of Nuclear Medicine, Odense University Hospital, 5000 Odense C, Denmark

**Correspondence to:**
Manouchehr Seyedi Vafaee

University of Southern Denmark, J.B. Winsløws vej 18, 5000 Odense C, Denmark

E-mail: [mvafaee@health.sdu.dk](mailto:mvafaee@health.sdu.dk) **Online Resource 4.** Specifics and details concerning the PET analysis.

Region-of-interest (ROI) analysis was conducted using PMOD software (PMOD Technologies Ltd., Version 4.4). The PNEURO tool facilitated segmentation of the T1-weighted MRI image into gray matter (GM), white matter (WM), and cerebrospinal fluid (CSF). PNEURO performed PET and MRI matching, motion correction, normalization using tissue probability maps, delineation of brain areas, and ROI analysis. The automated anatomical atlas 3 (AAL 3) (1) was the basis for this analysis. We generated a global mask, comprising 96 GM ROIs, and a composite mask consisting of 16 unilateral regions associated with early Aβ-deposition (See Supplementary Table 2 for overview of brain regions) (2,3). Additionally, the ROI analysis included the bilateral posterior cingulate cortex (PC), mid cingulate (MC), anterior cingulate (AC), precuneus, orbitofrontal cortex (OFC), paracentral lobule, insula, precentral gyrus, and parahippocampus. These regions were included because they are associated with early Aβ-deposition (2,3). The hippocampus was also included, given its involvement in AD. Subsequently, unilateral time-activity curves (TAC) were extracted from the ROIs, and from the cerebellum. Regional FDG- and PiB-standardized uptake value ratio (SUVR) ROI measures were then generated by averaging the unilateral TACs between 40-60 min for FDG and 40-70 min for PiB and dividing them by the cerebellar GM TAC. The PiB analysis involved 80 participants with an average age of 50.52 ± 17.88, including 35 males. In contrast, the FDG analysis comprised 78 individuals with a mean age of 50.92 ± 17.70, including 35 males. VoxelStats (4) was utilized to explore the voxel-wise relationship between PiB- and FDG-SUVR, while adjusting for age and gender. After motion correction using FSLs MCFLIRT (5), the mean of the 40-70 min PiB scans and the 40-60 min FDG scans were affine registered to the T1-weighted image and non-linearly transformed to Montreal Neurological Institute (MNI) space using FSLs FNIRT (6). Subsequently images were normalized by the cerebellar GM to generate SUVR images. These images underwent smoothing with a 4 mm full width half maximum (FWHM) Gaussian kernel. Only subjects with both a successful PiB and a FDG scan were included in the analysis (n = 78, mean age 50.92 ± 17.70, 35 males). The analysis was restricted to GM using FSL's MNI average152 tissue prior gray mask thresholded at 40% probability. Results obtained from the linear model underwent correction for cluster-based multiple comparison correction and were visualized using MRIcroGL (7) and surf ice software (<https://www.nitrc.org/projects/surfice/>).

1. Rolls ET, Huang CC, Lin CP, Feng J, Joliot M. Automated anatomical labelling atlas 3. Neuroimage. 2020;206(September 2019):116189.

2. Collij LE, Heeman F, Salvadó G, Ingala S, Altomare D, De Wilde A, et al. Multitracer model for staging cortical amyloid deposition using PET imaging. Neurology. 2020;95(11):E1538–53.

3. Mattsson N, Palmqvist S, Stomrud E, Vogel J, Hansson O. Staging β -Amyloid Pathology with Amyloid Positron Emission Tomography. JAMA Neurol. 2019;76(11):1319–29.

4. Mathotaarachchi S, Wang S, Shin M, Pascoal TA, Benedet AL, Kang MS, et al. VoxelStats: A MATLAB package for multi-modal voxel-wise brain image analysis. Front Neuroinform. 2016;10(JUNE):1–12.

5. Smith SM, Jenkinson M, Woolrich MW, Beckmann CF, Behrens TEJ, Johansen-Berg H, et al. Advances in functional and structural MR image analysis and implementation as FSL. Neuroimage. 2004;23(SUPPL. 1):208–19.

6. Jenkinson M, Beckmann CF, Behrens TEJ, Woolrich MW, Smith SM. Review FSL. Neuroimage. 2012;62:782–90.

7. Rorden C, Bonilha L, Fridriksson J, Bender B, Karnath HO. Age-specific CT and MRI templates for spatial normalization. Neuroimage. 2012;61(4):957–65.
