# Supplementary material for: Multimodal neuroimaging insights into the neurobiology of healthy aging across the lifespan
Source: Eur J Nucl Med Mol Imaging. 2025 Feb 1;52(7):2267–78. doi: 10.1007/s00259-025-07100-w (PMC12119650; doi:10.1007/s00259-025-07100-w)
Supplement: Supplementary file 5 — Supplementary Material 5 [file 259_2025_7100_MOESM5_ESM.docx]

**Multimodal Neuroimaging Insights into the Neurobiology of Healthy Aging Across the Lifespan**

European Journal of Nuclear Medicine and Molecular Imaging

Laust Vind Knudsen^1^, Tanja Maria Michel^1^**^†^**, Ziba Ahangarani Farahani^2^, Manouchehr Seyedi Vafaee^1,2^

**^†^**Shared first author

**Author affiliations:**

^1^ Department of Psychiatry, University of Southern Denmark, 5000 Odense C, Denmark

^2^ Department of Nuclear Medicine, Odense University Hospital, 5000 Odense C, Denmark

**Correspondence to:**
Manouchehr Seyedi Vafaee

University of Southern Denmark, J.B. Winsløws vej 18, 5000 Odense C, Denmark

E-mail: [mvafaee@health.sdu.dk](mailto:mvafaee@health.sdu.dk) **Online Resource 5.** Specifics and details concerning the MRI analysis.

After visual inspection 71 data sets were included in the fMRI analysis (mean age 52.72 ± 17.35, 29 male). The data underwent preprocessing and analysis using the Functional Connectivity Toolbox (CONN version 22.a) in MATLAB R2021b.(1) A default CONN preprocessing pipeline was employed, encompassing functional realignment and unwarping, slice timing correction, direct segmentation, and normalization to MNI space, co-registration to the reference image, identification of potential outlier scans, and smoothing with a Gaussian kernel of 8 mm FWHM. For denoising, physiological and motion-related noise correction was conducted using CompCor, and frequency filtering between 0.008-0.09 Hz. Outlier scans resulting from motion were detected using the Artifact Rejection Toolbox, with the 97th percentile as the threshold. Invalid scans were defined as a BOLD signal deviation of ± 5 SD from the mean global signal or a framewise displacement of at least 0.9 mm and were subsequently scrubbed during the denoising process. Dynamic functional connectivity (dFC) analysis was performed using a 100s sliding-window length and a 25s separation between windows. ROI-to-ROI-based analysis was performed using the Consensual Atlas of Resting-state Network (CAREN).(2) Specifically, we investigated the effect of default-mode-network (DMN)(3) PiB-SUVR on dFC variability while correcting for age, gender, and mean motion. DFC represents the degree of temporal variability in functional connectivity (FC) between pairs of ROIs. The DMN regions previously involved in the PET analysis were used as seed-region, including the AC, insula, MC, OFC, parahippocampus, PC, and precuneus. False discovery rate (FDR) corrected p-values below 0.05 were considered significant. Next, all DMN regions in the CAREN atlas were used for an exploratory ROI-to-ROI analysis. Threshold-free-cluster-enhancement was used for inference with a FDR-corrected p-value below 0.05 considered significant. Subsequently, a graph-theory analysis was conducted, encompassing all DMN regions in the CAREN atlas, to evaluate the clustering coefficient. The analysis utilized default settings, and significance was determined based on FDR-corrected p-values below 0.05. The analysis of the ASL data (n = 60, mean age 52.84 ± 16.53, 27 males) followed the procedure outlined by Zhao *et al*.(4) In summary, the ASL data were quantified using model-fitting and spatial Bayesian inference methods implemented in the FMRIB Software Library (FSL) v6.0, specifically through the Bayesian Inference for Arterial Spin Labeling MRI (BASIL) tool.(5) CBF quantification involved utilizing a proton density calibration image (M0), with signal intensity corrected for a blood-water partition coefficient of 0.9. Furthermore, noise reduction of the calibration image was executed through a median filter and erosion of the brain edge. The preprocessed calibration image was then employed for CBF quantification, assuming a labeling efficiency of 0.85,(6) facilitating the expression of CBF in absolute units (mL/min/100g). Subsequently the data were transformed from structural T1-weighted space to MNI standard space. Permutation inference was conducted utilizing FSL's randomization and threshold-free cluster enhancement (TFCE). (7) This analysis aimed to explore the impact of age and PiB-SUVR on CBF, with gender and both gender and age used as covariates, respectively. Tract-Based-Spatial-Statistics (TBSS) from FSL were employed for the dMRI analysis (n = 65, mean age 52.25 ± 17.27, 29 males). The selection of the most representative image, i.e., the one requiring minimal warping for alignment with others, was carried out to enhance the registration procedure.(8) Following this, statistical testing on fractional anisotropy (FA) images was conducted using FSL's randomize, with 2-dimensional TFCE utilized for optimization purposes.(8) FA is a measure of WM integrity, where lower FA is interpreted as less organized and potentially damaged WM. The objective of this analysis was initially to investigate the influence of age with gender as covariate and subsequently to test the influence of PiB-SUVR on FA with both gender and age as covariates.

1. Nieto-Castanon A, Whitfield-Gabrieli S. CONN functional connectivity toolbox: RRID SCR_009550, release 22. CONN functional connectivity toolbox: RRID SCR_009550, release 22. 2022;

2. Doucet GE, Lee WH, Frangou S. Evaluation of the spatial variability in the major resting-state networks across human brain functional atlases. Hum Brain Mapp. 2019;40(15):4577–87.

3. Raichle ME, MacLeod AM, Snyder AZ, Powers WJ, Gusnard DA, Shulman GL. A default mode of brain function. Proc Natl Acad Sci U S A. 2001;98(2):676–82.

4. Zhao MY, Mezue Melvin, Segerdahl AR, W okell T, Tracey I, Xiao Y, et al. A Systematic Study of the Sensitivity of Partial Volume Correction Methods for the Quantification of Perfusion from Pseudo-continuous Arterial Spin Labeling MRI. Neuroimage. 2017;(162):384–97.

5. Chappell MA, Groves AR, Whitcher B, Woolrich MW. Variational Bayesian inference for a nonlinear forward model. IEEE Transactions on Signal Processing. 2009;57(1):223–36.

6. Dai W, Garcia D, De Bazelaire C, Alsop DC. Continuous flow-driven inversion for arterial spin labeling using pulsed radio frequency and gradient fields. Magn Reson Med. 2008;60(6):1488–97.

7. Winkler AM, Ridgway GR, Webster MA, Smith SM, Nichols TE. Permutation inference for the general linear model. Neuroimage. 2014;92:381–97.

8. Smith SM, Jenkinson M, Johansen-Berg H, Rueckert D, Nichols TE, Mackay CE, et al. Tract-based spatial statistics: Voxelwise analysis of multi-subject diffusion data. Neuroimage. 2006;31(4):1487–505.
