# Supplementary material for: Multimodal neuroimaging insights into the neurobiology of healthy aging across the lifespan
Source: Eur J Nucl Med Mol Imaging. 2025 Feb 1;52(7):2267–78. doi: 10.1007/s00259-025-07100-w (PMC12119650; doi:10.1007/s00259-025-07100-w)
Supplement: Supplementary file 6 — Supplementary Material 6 [file 259_2025_7100_MOESM6_ESM.docx]

**Multimodal Neuroimaging Insights into the Neurobiology of Healthy Aging Across the Lifespan**

European Journal of Nuclear Medicine and Molecular Imaging

Laust Vind Knudsen^1^, Tanja Maria Michel^1^**^†^**, Ziba Ahangarani Farahani^2^, Manouchehr Seyedi Vafaee^1,2^

**^†^**Shared first author

**Author affiliations:**

^1^ Department of Psychiatry, University of Southern Denmark, 5000 Odense C, Denmark

^2^ Department of Nuclear Medicine, Odense University Hospital, 5000 Odense C, Denmark

**Correspondence to:**
Manouchehr Seyedi Vafaee

University of Southern Denmark, J.B. Winsløws vej 18, 5000 Odense C, Denmark

E-mail: [mvafaee@health.sdu.dk](mailto:mvafaee@health.sdu.dk) **Online Resource 6.** Kolmogorov-Smirnov test for normality. P-values below 0.05 indicate non-normality. The tables demonstrate that 14 of 24 regions were not normally distributed.

| **Region** | **PiB** | **FDG** |
| --- | --- | --- |
| Anterior cingulate | 0.0021 | >0.1000 |
| Composite | 0.0093 | 0.0266 |
| Global | 0.0031 | 0.0122 |
| Hippocampus | >0.1000 | >0.0100 |
| Insula | <0.0001 | 0.0281 |
| Mid cingulate | <0.0001 | 0,0096 |
| Orbitofrontal cortex | >0.1000 | >0.0100 |
| Paracentral lobule | 0.0398 | >0.0100 |
| Parahippocampus | <0.1000 | >0.0100 |
| Posterior cingulate | <0.0001 | 0.0283 |
| Precentral gyrus | 0.0004 | >0.0100 |
| Precuneus | <0.0001 | >0.0100 |
