# Supplementary material for: Multimodal neuroimaging insights into the neurobiology of healthy aging across the lifespan
Source: Eur J Nucl Med Mol Imaging. 2025 Feb 1;52(7):2267–78. doi: 10.1007/s00259-025-07100-w (PMC12119650; doi:10.1007/s00259-025-07100-w)
Supplement: Supplementary file 7 — Supplementary Material 7 [file 259_2025_7100_MOESM7_ESM.docx]

**Multimodal Neuroimaging Insights into the Neurobiology of Healthy Aging Across the Lifespan**

European Journal of Nuclear Medicine and Molecular Imaging

Laust Vind Knudsen^1^, Tanja Maria Michel^1^**^†^**, Ziba Ahangarani Farahani^2^, Manouchehr Seyedi Vafaee^1,2^

**^†^**Shared first author

**Author affiliations:**

^1^ Department of Psychiatry, University of Southern Denmark, 5000 Odense C, Denmark

^2^ Department of Nuclear Medicine, Odense University Hospital, 5000 Odense C, Denmark

**Correspondence to:**
Manouchehr Seyedi Vafaee

University of Southern Denmark, J.B. Winsløws vej 18, 5000 Odense C, Denmark

E-mail: [mvafaee@health.sdu.dk](mailto:mvafaee@health.sdu.dk)

**Online Resource 7.** Results from the ROI-to-ROI seed-based analysis including only ROIs from the initial PiB and FDG analysis. Results exhibit regions that had significant correlation between dFC variability and PiB-SUVR of the DMN corrected for age, gender, and mean motion.

| **Seed-region** | **Region of change** | **Mean** | **Range** | **Beta** | **T** | **p-FDR** |  |
| --- | --- | --- | --- | --- | --- | --- | --- |
| ACC_Sub_L | Occipital_mid_L | 0.193 | 0.10-0.33 | 0.27 | 3.83 | 0.018321 | |
| ACC_Sup_L | ACC_sup_R | 0.202 | 0.09-0.39 | 0.34 | 3.98 | 0.011291 | |
| ACC_Sup_L | Lingual_R | 0.209 | 0.08-0.47 | 0.29 | 3.58 | 0.020541 | |
| Insula R | Temporal_Pole_sup_L | 0.196 | 0.08-0.51 | 0.38 | 4.93 | 0.000379 | |
| Insula R | OFC_post_L | 0.193 | 0.09-0.40 | 0.22 | 3.68 | 0.015203 | |
| Insula R | Cuneus_L | 0.186 | 0.09-0.41 | 0.23 | 3.36 | 0.027885 | |
| OFC_Ant_R | Frontal_inf_orb_R | 0.199 | 0.11-0.38 | 0.37 | 4.32 | 0.003490 | |
| Parahippocampus_L | SupraMarginal_L | 0.190 | 0.09-0.37 | 0.30 | 3.80 | 0.020015 | |
