# Supplementary material for: Multimodal neuroimaging insights into the neurobiology of healthy aging across the lifespan
Source: Eur J Nucl Med Mol Imaging. 2025 Feb 1;52(7):2267–78. doi: 10.1007/s00259-025-07100-w (PMC12119650; doi:10.1007/s00259-025-07100-w)
Supplement: Supplementary file 8 — Supplementary Material 8 [file 259_2025_7100_MOESM8_ESM.docx]

**Multimodal Neuroimaging Insights into the Neurobiology of Healthy Aging Across the Lifespan**

European Journal of Nuclear Medicine and Molecular Imaging

Laust Vind Knudsen^1^, Tanja Maria Michel^1^**^†^**, Ziba Ahangarani Farahani^2^, Manouchehr Seyedi Vafaee^1,2^

**^†^**Shared first author

**Author affiliations:**

^1^ Department of Psychiatry, University of Southern Denmark, 5000 Odense C, Denmark

^2^ Department of Nuclear Medicine, Odense University Hospital, 5000 Odense C, Denmark

**Correspondence to:**
Manouchehr Seyedi Vafaee

University of Southern Denmark, J.B. Winsløws vej 18, 5000 Odense C, Denmark

E-mail: [mvafaee@health.sdu.dk](mailto:mvafaee@health.sdu.dk)

**Online Resource 8.** Results from the ROI-to-ROI fMRI analysis including all regions of the CAREN DMN atlas. Significant p-value signifies an association between dFC variability and PiB-SUVR of the DMN corrected for age, gender, and mean motion.

| **Analysis unit** | **Statistic** | **p-uncorrected** | | **p-FDR** | |
| --- | --- | --- | --- | --- | --- |
| Cluster 1/1 | TFCE = 40.36 | 0.000075 | 0.024776 | |  |
|  |  |  |  |  |  |
| **Connection (in cluster)** | **T** | **p-uncorrected** | **p-FDR** | |  |
| Insula_R to Temporal_pole_Sup_L | 4.93 | 0.000006 | 0.12307 | |  |
| Temporal_sup_R to Temporal_pole_Sup_L | 1.78 | 0.080347 | 0.995828 | |  |
| Temporal_sup_R to OFC_post_R | 1.35 | 0.180111 | 0.995828 | |  |
| Temporal_sup_R to Rectus_L | 1.17 | 0.246863 | 0.995828 | |  |

TFCE = Threshold-free-cluster-enhancement
