# Supplementary material for: Multimodal neuroimaging insights into the neurobiology of healthy aging across the lifespan
Source: Eur J Nucl Med Mol Imaging. 2025 Feb 1;52(7):2267–78. doi: 10.1007/s00259-025-07100-w (PMC12119650; doi:10.1007/s00259-025-07100-w)
Supplement: Supplementary file 9 — Supplementary Material 9 [file 259_2025_7100_MOESM9_ESM.docx]

**Multimodal Neuroimaging Insights into the Neurobiology of Healthy Aging Across the Lifespan**

European Journal of Nuclear Medicine and Molecular Imaging

Laust Vind Knudsen^1^, Tanja Maria Michel^1^**^†^**, Ziba Ahangarani Farahani^2^, Manouchehr Seyedi Vafaee^1,2^

**^†^**Shared first author

**Author affiliations:**

^1^ Department of Psychiatry, University of Southern Denmark, 5000 Odense C, Denmark

^2^ Department of Nuclear Medicine, Odense University Hospital, 5000 Odense C, Denmark

**Correspondence to:**
Manouchehr Seyedi Vafaee

University of Southern Denmark, J.B. Winsløws vej 18, 5000 Odense C, Denmark

E-mail: [mvafaee@health.sdu.dk](mailto:mvafaee@health.sdu.dk)

**Online Resource 9.** Results from the graph-theory analysis using the measure of clustering coefficient. All regions of the CAREN DMN atlas were included in the analysis. Only the left insula demonstrated significant association between clustering coefficient and DMN PiB SUVR. Analysis threshold was 0.05 p-FDR corrected.

| **Seed-region** | **Region of change** | **Beta** | **T** | **p-uncorrected** | | **p-FDR** |
| --- | --- | --- | --- | --- | --- | --- |
| Insula_L | CAREN_DMN | 0.73 | 4.75 | | 0.000012 | 0.000765 |
