# Supplementary material for: Multimodal neuroimaging insights into the neurobiology of healthy aging across the lifespan
Source: Eur J Nucl Med Mol Imaging. 2025 Feb 1;52(7):2267–78. doi: 10.1007/s00259-025-07100-w (PMC12119650; doi:10.1007/s00259-025-07100-w)
Supplement: Supplementary file 10 — Supplementary Material 10 [file 259_2025_7100_MOESM10_ESM.docx]

**Multimodal Neuroimaging Insights into the Neurobiology of Healthy Aging Across the Lifespan**

European Journal of Nuclear Medicine and Molecular Imaging

Laust Vind Knudsen^1^, Tanja Maria Michel^1^**^†^**, Ziba Ahangarani Farahani^2^, Manouchehr Seyedi Vafaee^1,2^

**^†^**Shared first author

**Author affiliations:**

^1^ Department of Psychiatry, University of Southern Denmark, 5000 Odense C, Denmark

^2^ Department of Nuclear Medicine, Odense University Hospital, 5000 Odense C, Denmark

**Correspondence to:**
Manouchehr Seyedi Vafaee

University of Southern Denmark, J.B. Winsløws vej 18, 5000 Odense C, Denmark

E-mail: [mvafaee@health.sdu.dk](mailto:mvafaee@health.sdu.dk)

**Online Resource 10.** Results from the fractional anisotropy analysis.


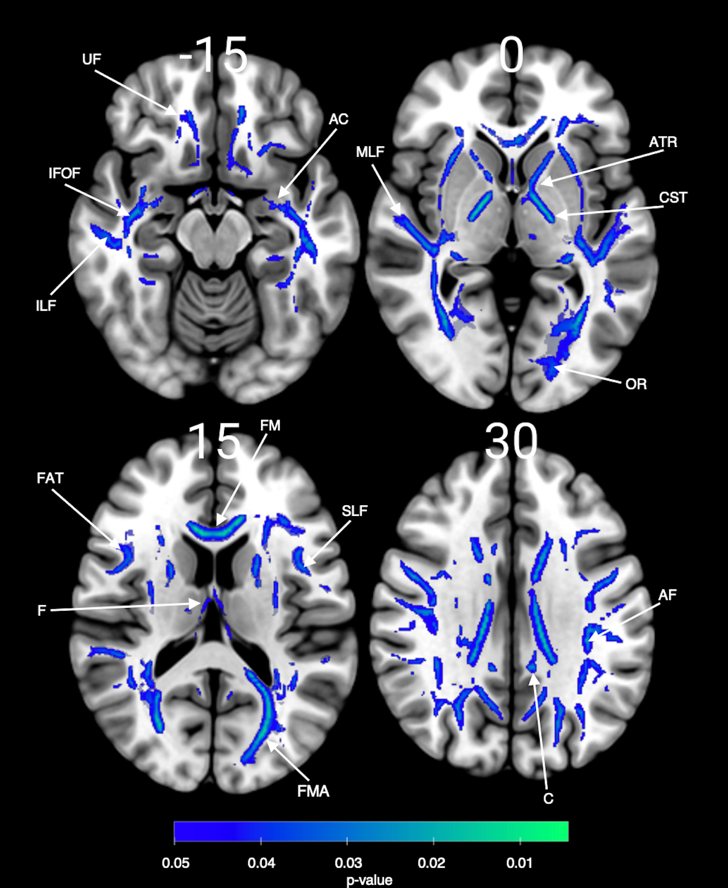


The statistically significant maps, corrected for multiple comparisons and adjusted for gender, are displayed in axial slices, and overlaid on the MNI152 image. The blue-green color highlights areas where fractional anisotropy decreases with advancing age. Tracts specification is based on the XTRACT HCP probabilistic Tract Atlases. Inferior longitudinal fasciculus = ILF. Inferior Fronto-Occipital Fasciculus = IFOF. Uncinate Fasciculus = UF. Middle longitudinal fasciculus = MLF. Anterior commissure = AC. Optic Radiation = OR. Anterior Thalamic radiation = ATR. Forceps Minor = FM. Corticospinal tract = CST. Forceps Major = FMA. Fornix = F. Frontal Aslant tract = FAT. Superior longitudinal fasciculus = SLF. Arcuate Fasciculus = AF. Cingulum dorsal = C.
