# Supplementary material for: Multimodal neuroimaging insights into the neurobiology of healthy aging across the lifespan
Source: Eur J Nucl Med Mol Imaging. 2025 Feb 1;52(7):2267–78. doi: 10.1007/s00259-025-07100-w (PMC12119650; doi:10.1007/s00259-025-07100-w)
Supplement: Supplementary file 11 — Supplementary Material 11 [file 259_2025_7100_MOESM11_ESM.docx]

**Multimodal Neuroimaging Insights into the Neurobiology of Healthy Aging Across the Lifespan**

European Journal of Nuclear Medicine and Molecular Imaging

Laust Vind Knudsen^1^, Tanja Maria Michel^1^**^†^**, Ziba Ahangarani Farahani^2^, Manouchehr Seyedi Vafaee^1,2^

**^†^**Shared first author

**Author affiliations:**

^1^ Department of Psychiatry, University of Southern Denmark, 5000 Odense C, Denmark

^2^ Department of Nuclear Medicine, Odense University Hospital, 5000 Odense C, Denmark

**Correspondence to:**
Manouchehr Seyedi Vafaee

University of Southern Denmark, J.B. Winsløws vej 18, 5000 Odense C, Denmark

E-mail: [mvafaee@health.sdu.dk](mailto:mvafaee@health.sdu.dk) **Online Resource 11.** Results from the ASL analysis

O

R


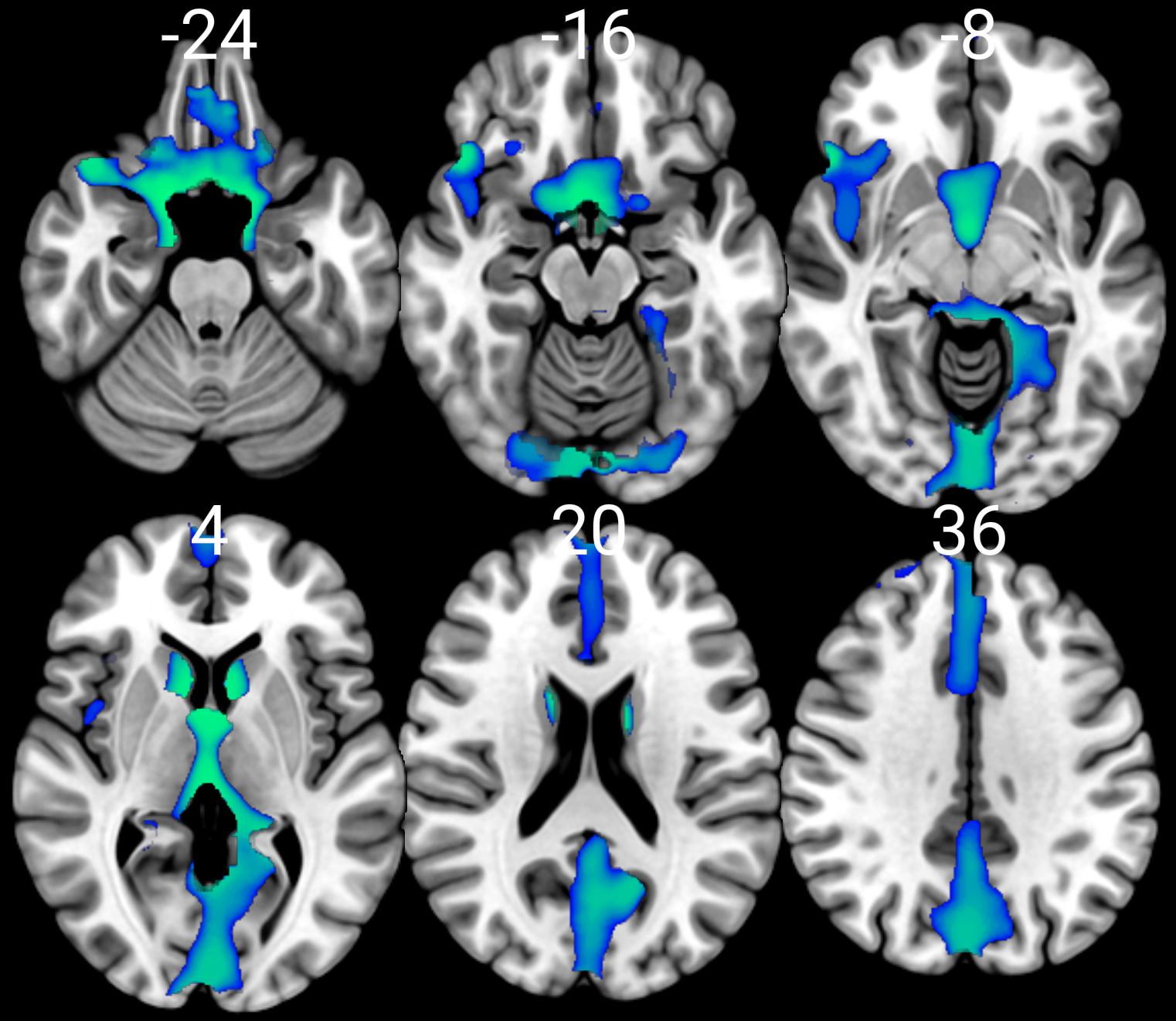


FSO

FIO

I

CA

TPS

PH

FF

FSM

C

L

FS

AC

MC

T

H

PRE

CU


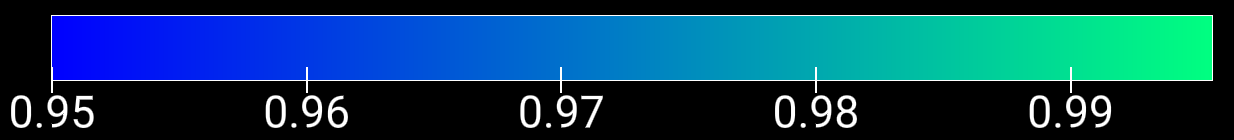


The axial slices display statistically significant maps from the arterial spin labeling analysis, adjusted for gender and corrected for multiple comparisons, overlaid on the MNI152 image. Areas where cerebral blood flow decreases with advancing age are highlighted in blue-green. Temporal Pole superior = TPS. Frontal superior orbital = FSO. Frontal inferior orbital = FIO. Rectus = R. Parahippocampal = PH. Fusiform = FF. Lingual = L. Insula = I. Caudate = CA. Calcarine fissure = C. Frontal superior medial = FSM. Thalamus = T. Hippocampus = H. Anterior Cingulate = AC. Precuneus = PRE. Cuneus = CU. Frontal superior = FS. Cingulate mid = MC.
